# Randomized Phase II Study of 5-Fluorouracil Hepatic Arterial Infusion with or without Antineoplastons as an Adjuvant Therapy after Hepatectomy for Liver Metastases from Colorectal Cancer

**DOI:** 10.1371/journal.pone.0120064

**Published:** 2015-03-19

**Authors:** Yutaka Ogata, Keiko Matono, Hideaki Tsuda, Masataka Ushijima, Shinji Uchida, Yoshito Akagi, Kazuo Shirouzu

**Affiliations:** 1 Department of Surgery, Kurume University Medical Center, Kurume, Japan; 2 Departments of Surgery, Kurume University School of Medicine, Kurume, Japan; 3 Kurume Daiichi Social Insurance Hospital, Kurume, Japan; Davidoff Center, ISRAEL

## Abstract

**Background:**

Antineoplastons are naturally occurring peptides and amino acid derivatives found in human blood and urine. Antineoplaston A10 and AS2-1 reportedly control neoplastic growth and do not significantly inhibit normal cell growth. Antineoplastons contain 3-phenylacetylamino-2, 6-piperidinedione (A10), phenylacetylglutamine plus phenylacetylisoglutamine (A10-I), and phenylacetylglutamine plus phenylacetate (AS2-1). This open label, non- blinded randomized phase II study compared the efficacy of hepatic arterial infusion (HAI) with 5-fluorouracil,with or without antineoplastons as a postoperative therapy for colorectal metastasis to the liver.

**Methods:**

Sixty-five patients with histologically confirmed metastatic colon adenocarcinoma in liver, who had undergone hepatectomy, and/or thermal ablation for liver metastases were enrolled between 1998- 2004 in Kurume University Hospital. Patients were randomly assigned to receive systemic antineoplastons (A10-I infusion followed by per-oral AS2-1) plus HAI (AN arm) or HAI alone (control arm) based on the number of metastases and presence/ absence of extra-hepatic metastasis at the time of surgery. Primary endpoint was cancer-specific survival (CSS); secondary endpoints were relapse-free survival (RFS), status and extent of recurrence, salvage surgery (rate) and toxicity.

**Findings:**

Overall survival was not statistically improved (p=0.105) in the AN arm (n=32). RFS was not significant (p=0.343). Nevertheless, the CSS rate was significantly higher in the AN arm versus the control arm (n=33) with a median survival time 67 months (95%CI 43-not calculated) versus 39 months (95%CI 28-47) (p=0.037) and 5 year CSS rate 60% versus 32% respectively. Cancer recurred more often in a single organ than in multiple organs in the AN arm versus the control arm. The limited extent of recurrent tumours in the AN arm meant more patients remained eligible for salvage surgery. Major adverse effects of antineoplastons were fullness of the stomach and phlebitis. No serious toxicity, including bone marrow suppression, liver or renal dysfunction, were found in the AN arm.

**Interpretation:**

Antineoplastons (A10 Injection and AS2-1) might be useful as adjunctive therapy in addition to HAI after hepatectomy in colorectal metastases to the liver.

**Trial registration information:**

ClinicalTrials.gov UMIN000012099

## Introduction

A recent meta-analysis which reviewed studies published between 1999 and 2010 revealed a 5- and 10-year-survival rate for patients undergoing resection of liver metastases of 16–74% (median 38%) and 9–69% (median 26%) with median overall survival time of 3.6 years (median 42 months) [1]. It has been reported that long-term survival following liver resection for colorectal metastases has improved significantly in recent years [2].

However, up to 70% of patients will have disease recurrence following resection [1,3]. Systemic chemotherapy alone in the adjuvant setting after liver resection has been evaluated in several studies [4]. A meta-analysis showed an increase in disease-free survival (DFS) with the use of systemic 5-fluorouracil (5-FU) and leucovorin, but only after adjustment for poor prognostic factors [5]. Systemic FU/leucovorin/oxaliplatin (FOLFOX) both before and after liver metastatic tumor resection demonstrated a significant increase in progression-free survival (PFS) compared with no systemic treatment [6], and the difference in PFS was not significant in the intention-to-treat population of the study.

Hepatic arterial infusion of chemotherapy (HAI using 5-FU) has been shown to increase response rates when treating liver metastases, but the overall survival benefit attributed to HAI with chemotherapy is minimal or negligible due to extra-hepatic recurrence in unresectable liver metastases [7]. Postoperative HAI alone after liver resection reduces hepatic recurrence but shows no survival benefit [8]. HAI is thought to be less effective for extra-hepatic tumors because 5-FU is deactivated in the liver circulation [9].

As neither systemic chemotherapy nor HAI alone seems to be effective in improving survival after liver resection, the combination of HAI and systemic chemotherapy to control intra-hepatic recurrence and extra-hepatic recurrence, in particular lung metastasis, becomes an important approach [10, 11].

Antineoplastons are naturally occurring peptides and amino acid derivatives found in human blood and urine, first described by Burzynski in 1976 [12]. Antineoplaston A10 (3-phenylacetylamino-2, 6-piperidinedione) was the first chemically-identified antineoplaston. When administered per-orally it yields partially phenylacetylglutamine (PG) and phenylacetylisoglutamine (isoPG) by hydrolysis in the pancreatic juice. PG and isoPG are further metabolized to phenylacetate (PA) in liver. The mixture of PG and isoPG (ratio of 4:1) is formulated as antineoplaston A10 Injection (A10-I). The mixture of PG and PA (ratio of 1:4) has been formulated as antineoplaston AS2–1. Antineoplaston A10 and AS2–1 have been found to control neoplastic growth in tissue culture study with hepatocellular carcinoma cell-lines [13,14] and in an animal study with transplanted human breast cancer [15]. Our phase I clinical toxicological study [16] demonstrated the minimal adverse effects of these agents. Thus, it is postulated that combining these antineoplastons with 5-FU HAI would have the potential to increase antitumor effect without decreasing the patient’s quality of life. The antitumor effect of antineoplastons on micrometastases when used as an adjuvant in the experimental lung metastasis model of orthotopically implanted colon cancer in nude rats was confirmed in our study [17].

In this randomized phase II clinical study, we investigated the antitumor effects and toxicity of antineoplastons (A10 Injection and AS2–1) in addition to 5-FU HAI after hepatectomy in colorectal metastases to the liver.

## Patients and Methods

The protocol for this trial and supporting CONSORT checklist are available as supporting information; see S1 CONSORT Checklist and S1 Protocol. Eligible patients had histologically confirmed metastatic colorectal adenocarcinoma to the liver. Patients were treated with R0 resection of liver metastases and/or complete ablation by radio frequency interstitial ablation therapy between April 1998 and August 2004 at Kurume University Hospital. The inclusion criteria were age ≤75 years, no severe major organ dysfunction, no prior cancer therapy to the liver, no extra-hepatic metastases at study entry, Eastern Cooperative Oncology Group (ECOG) performance status ≤2, no other malignancy (within the 5 years prior to study entry), white blood cell count ≥3000/μL, absolute neutrophil count ≥1500/μL, platelet count ≥75,000/μL, hemoglobin ≥ 10 g/dL, AST/ALT ≤ 100 IU, creatinine ≤ 1.5 mg/dL and total bilirubin ≤ 2.0 mg/dL. Computed tomography (CT) scans of the chest, abdomen, and pelvis were required to have been carried out within the 6 weeks prior to protocol registration. All patients provided signed informed consent after hepatectomy; the protocol and informed consent were approved by the Kurume University School of Medicine Institutional Review Board and registered with study number 9717. The study was performed in accordance with the Declaration of Helsinki, with local ethics committee approval. This clinical study was registered with ID number of UMIN000012099 on the date of October 22th 2013. It was not registered previously because enrollment for this study finished in 2003 before the start of mandatory official registration for clinical trials in Japan (UMIN- CTR) in 2006. The authors confirm that all ongoing and related trials for this drug are registered. This study was started in 1998 and the results were confirmed by comparing cancer-specific survival (CSS) [18], relapse-free survival (RFS) and salvage surgery rate in 10 years.

### Randomization and study design

Eligible patients were randomly assigned to receive systemic antineoplastons plus 5-FU HAI (AN arm) or 5-FU HAI alone (control arm) by a minimization method using number of metastases (1–3 *vs* ≥4) and presence or absence of prior extra-hepatic metastases which were removed completely (R0 resection) at the time of surgery. Randomization used 50:50 weighting to the two arms and was established by computed macro program in Microsoft Excel 97 (Microsoft Cooperation, Redmond, USA).

The study was an open-label, non- blinded randomized phase II screening design. The primary objective was to determine the efficacy and toxicity of systemic administration of antineoplastons as an adjuvant added to 5-FU HAI after liver resection. The primary endpoint was CSS and secondary endpoints were evaluation of RFS, status and extent of recurrence, salvage surgery (rate) and toxicity.

The efficacy of antineoplastons should be demonstrated in RFS, number of sites and extent of recurrence, salvage surgery rate and CSS since only patients with R0 resection (no visible tumor recognized) were enrolled at start of the study. Comparison with a randomized control group would be more convincing than historical comparisons because reported survival varies widely among institutes, studies and timing of trials.

### Treatment schedule

Patients received HAI with 5-FU (Kyowa Hakko Kirin, Tokyo, Japan) at a dose of 1000 mg/m^2^ for 4 hours weekly until the cumulative dose reached up to 15,000 mg. HAI with 2-week interval was infrequently allowed, when requested by the patients. Antineoplastons, A10-I and AS2–1, provided by Dr SR Burzynski (the patent holder of antineoplaston A10 and AS2–1, Burzynski Institute, Houston, TX, USA) were administered from day 15 after liver surgery. In the AN arm, a starting dose of 30 g/day of A10-I was administered intravenously (i.v.) using a pump system with maximum dose of 100 g/day for more than 3 days. After completion of the i.v. administration of A10-I for a week, 10 g/day of AS2–1 was administered orally for 1 year. Patients were required to have an absolute neutrophil count ≥ 1500/μL(HAI) and 1000/μL (antineoplastons), platelet count ≥75,000/μL, total bilirubin <2.0 mg/dL and creatinine ≤1.8 mg/dL for subsequent cycles of 5-FU HAI and antineoplastons (A10-I, AS2–1) to be administered. If counts were below these levels on the scheduled day, HAI was delayed and antineoplaston therapy was interrupted for 1 week.

### Assessment of toxicity

All toxicities were graded according to the National Cancer Institute Common Toxicity Criteria 2.0 (CTC 2.0). Liver function blood tests (LFT) were evaluated every 2 weeks during HAI. Changes in doses of 5-FU were calculated according to the changes in LFT. Because patients entered the study with various degrees of hepatic enzyme abnormalities, hepatic toxicity due to 5-FU treatment was defined as a significant increase over individual baseline values (two-fold or greater for alkaline phosphatase, three-fold or greater for aspartate aminotransferase or alanine aminotransferase, and a 1.5-fold or greater increase in bilirubin. Doses of 5-FU were lowered to 750 mg/m^2^ when hepatic toxicity or greater than grade 3 non-hematological adverse effects were observed. Epigastric pain or severe abdominal pain during HAI treatment resulted in the emptying of drug from the pump and evaluation of the source of the pain (including a repeat flow scan to rule out extra-hepatic perfusion).

### Follow-up

CT scans of the chest, abdomen, and pelvis, carcinoembryonic antigen levels, and LFT were performed every 3 months for the first 2 years, every 6 months for the next 3 years, and yearly after 5 years.

### Statistical analysis

At the time of the study’s design, published data suggested that 70% of patients who undergo resection of hepatic metastases have a recurrence within two years (50% of these recurrences are in the liver) and that the 2-year and 5-year survival rates for these patients is approximately 65% and 35%, respectively [3]. Hypothesizing based on a 5-year CSS after liver resection in HAI control arm of 35%, a sample size of 57 patients was estimated to screen an additive 20% in 5-year CSS in the AN arm with error levels alpha = 20% and beta = 20%. The decision to initiate a study would depend on additional factors including tolerability of the regimen. CSS was defined as time from liver resection to cancer death or last follow-up. Patients alive or deceased without recurrence were censored at the time of last follow-up. RFS was defined as time from liver resection to any recurrence or death, whichever occurred first. Survival curves were estimated using the Kaplan-Meier method and compared using the Breslow-Gehan-Wilcoxson test, by which test more weight could be placed on later events after disease recurrence, in particular, in CSS. Associations between categoric variables were assessed by using chi-square test or Fisher's exact test.

The treatment and assessment of outcomes had been conducted by the same investigating team through the trial with responsibility of principal investigator KS. YO was in charge of enrolling participants into trial as a coordinator of this study after eligibility was discussed by the team. There were no changes or deviations from protocol in treatment, intervention, outcomes and analysis.

## Results

### Patient demographics

**Fig 1:**
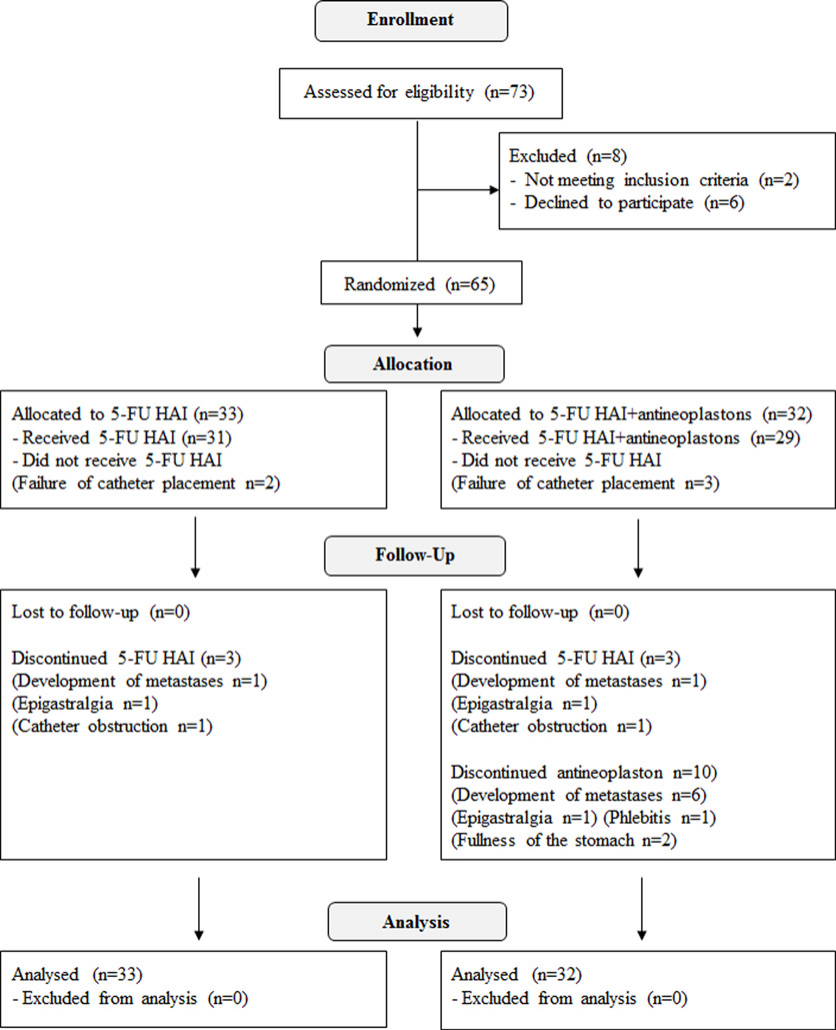
Patient disposition diagram.

Sixty-five patients who underwent liver resection according to the study inclusion criteria were entered into the study. The patients were to be randomly assigned to HAI with 5-FU with systemic antineoplaston therapy (32 patients) or 5-FU HAI therapy alone (33 patients) (Fig. 1). Patient characteristics are listed in Table 1. Established poor prognostic indicators [19] in the AN and control arms respectively, included lymph node involvement at the primary colorectal tumor site (72% *vs* 61%), multiple metastatic lesions (56% *vs* 55%), mean number of metastases (2.7 *vs* 2.7), lesion size >5 cm (13% *vs* 15%), <12-month interval from primary resection (78% *vs* 67%), treatment with concomitant radio frequency interstitial ablation therapy (34% *vs* 33%), and extra-hepatic metastasis (16% *vs* 18%). The Fong score was 2.2±0.8 in the AN arm and 2.0±1.2 in the control arm. The type of surgical procedure was similar in both arms with anatomical resection 16/32, 16/33; non-anatomical resection 14/32, 13/33; and ablation alone 2/32, 4/33; in the AN and control arms, respectively.

**Table 1:** Patients characteristics.

Arm	Age (years) (mean±sd)	Gender (M/F)	Lymph node involvement at primary tumor (%)	Multiple lesion (%)	Number of metastases (mean±sd)	Size>5cm (%)	< 12-month interval from primary surgery (%)	Concomitant ablation (RFA) (%)	Extra-hepatic Metastases (%)
AN (N = 32)	62.0±8.9	26/6	72	56	2.7±2.0	13	78	34	16
Control (N = 33)	60.0±10.2	21/12	61	55	2.7±2.2	15	67	33	18

RFA: radiofrequency ablation

### Toxicity

All abnormal values in testing and complaints (symptoms) during treatment were regarded as toxicity regardless of the abnormal base line value at the study entry. Toxicities are listed in Table 2. Adverse effects such as neutropenia, thrombocytopenia, anemia, AST/ALT abnormality, hyperbilirubinemia, increased creatinine, anorexia, nausea/vomiting, epigastralgia, fullness of stomach, stiffness of the finger and phlebitis were observed during treatment. There was no difference between arms in occurrence of those adverse effects except fullness of the stomach and phlebitis. Eight (25%) versus two (6%) patients developed fullness of the stomach (p = 0.044), and six (19%) versus zero developed phlebitis at the vessels used for A10 infusion (p<0.024) in the AN and control arms, respectively. These adverse effects seemed to be specific for antineoplaston therapy. There was no difference in grade of severity of each adverse effect between arms. The majority of patients completed the scheduled HAI therapy: 26 of 32 and 28 of 33 in the AN and control arms, respectively. The reasons for not completing HAI were failure of catheter placement (three and two patients), development of metastases (one and one patient), and complications such as epigastralgia and catheter obstruction (two and two patients), in the AN and control arms, respectively. No other adverse effects were found in the AN arm compared with the control arm. A total of ten patients did not complete the scheduled antineoplaston therapy. The reasons for not completing antineoplaston therapy were the development of metastases in six patients, epigastralgia in one, phlebitis in one, and fullness of the stomach in two patients, respectively (Fig. 1).

**Table 2:** Adverse effects.

Events	AN (N = 32)	Control (N = 33)	P-value	AN (N = 32)	Control (N = 33)	P-value
	All grade	All grade		Grade 3–4	Grade 3–4	
Neutropenia	7 (22%)	9 (27%)	NS	2 (6%)	3 (9%)	NS
Thrombocytopenia	2 (6%)	3 (9%)	NS	0	0	
Anemia	6 (19%)	7 (21%)	NS	1 (3%)	2 (6%)	NS
AST/ALT	12 (38%)	11 (33%)	NS	2 (6%)	2 (6%)	NS
Hyperbilirubinemia	6 (19%)	7 (21%)	NS	1 (3%)	1 (3%)	NS
Creatinine	2 (6%)	3 (9%)	NS	0	0	
Anorexia	8 (25%)	4 (12%)	NS	2 (6%)	1 (3%)	NS
Nausea/vomiting	4 (13%)	3 (9%)	NS	1 (3%)	0	NS
Epigastralgia	2 (6%)	1 (3%)	NS	1 (3%)	1 (3%)	NS
Fullness of the stomach	8 (25%)	2 (6%)	0.044	0	0	
Stiffness of the finger	3 (9%)	1 (3%)	NS	0	0	
Phlebitis	6 (19%)	0	0.024	-	-	

### Survival

Median follow-up period was 43 months for all patients and 120 months for survivors.

**Fig 2:**
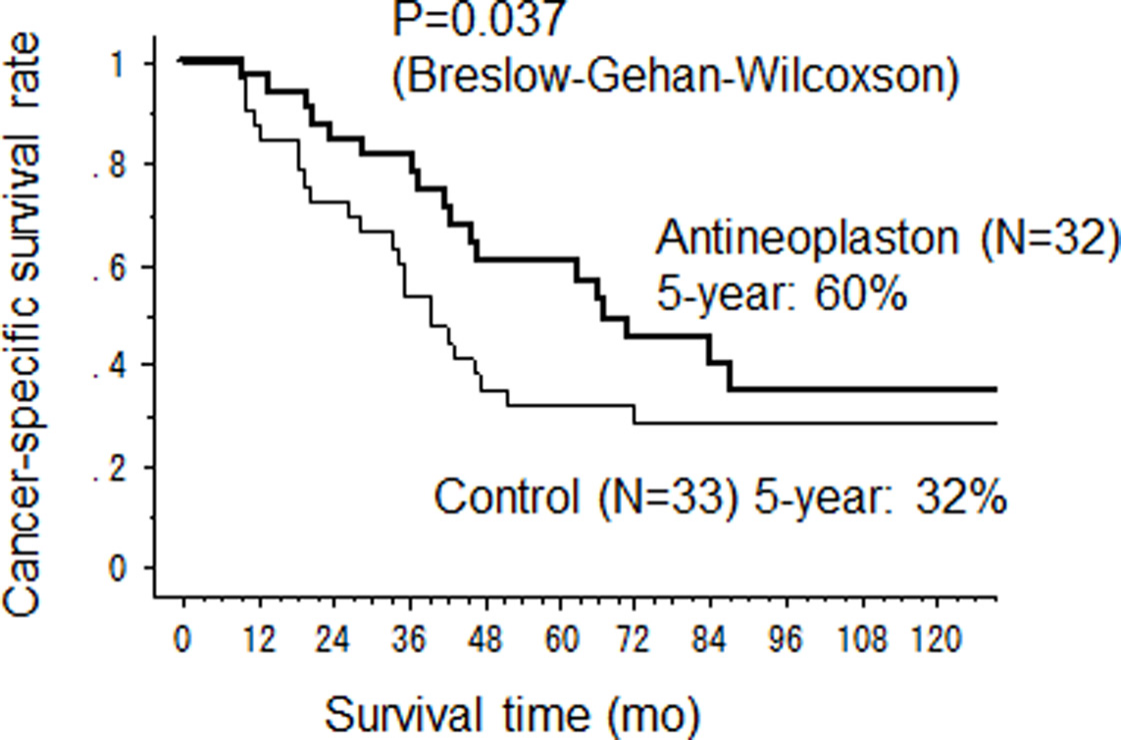
Cancer-specific survival after hepatectomy.

**Fig 3:**
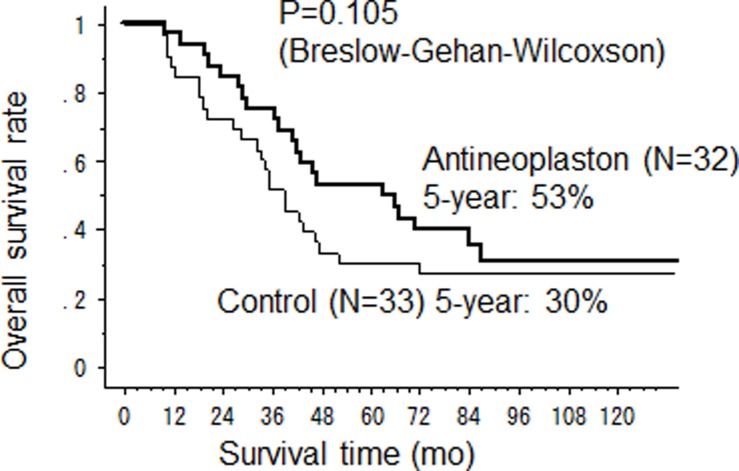
Overall survival after hepatectomy.

**Fig 4:**
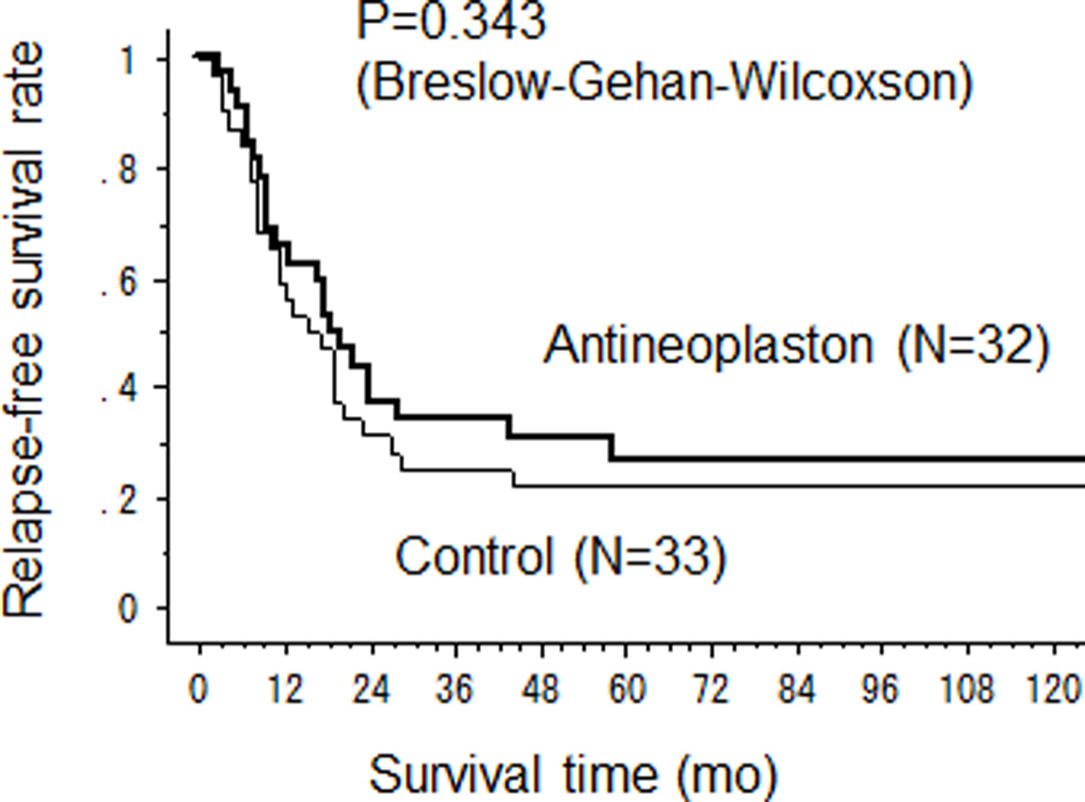
Relapse-free survival after hepatectomy.

The median CSS time in the AN arm was significantly longer than that in the control arm (67 months (95%CI 43-not calculated) versus 39 months (95%CI 28–47) (p = 0.037) (Fig. 2). The 2-year, 5-year and 10-year CSS rates were 84%, 60% and 35% in the AN arm and 73%, 32% and 29% in the control arm. The OS was also longer in the AN arm than that in the control arm with median OS time 64.5 months (95%CI 38–92) *vs* 39 months (95%CI 28–47), although no significant difference was observed (p = 0.105) (Fig. 3). Three patients died in AN arm from other causes, pneumonia, acute myocardial infarction, other cancer and there was one accidental death in the control arm. However, there was no significant difference in RFS between the AN and control arms with median RFS 18 months versus 16 months, respectively (Fig. 4).

### Post-operative recurrence and salvage surgical treatments

Post-operative recurrence occurred in 23 patients (72%) in the AN arm versus 26 patients (78%) in the control arm. The percentages of recurrence by site were liver; 44%, lung; 28% and other organs; 31%, in the AN arm, and in 55%, 42% and in 48% in the control arm, respectively. The recurrence rate in each organ in the AN arm tended to be lower than those in the control arm, although no significant difference was observed (Table 3). Of interest, there were numerically more patients whose disease had relapsed in multiple organs in the control arm compared with the AN arm (Table 4). Salvage surgery, including ablation therapy for recurrences at each organ site, could be carried out more frequently in the AN arm compared to the control arm (liver: 71% *vs* 44%; lung: 56% *vs* 21%; other organs 40% *vs* 31%), respectively (Table 3).

**Table 3:** Status of recurrence and salvage surgery.

Status of recurrence	AN		Control	
	Cases of recurrence (N = 23)	Cases of salvage surgery (N = 15)	Cases of recurrence (N = 26)	Cases of salvage surgery (N = 11)
Liver	14	10 (71%)	18	8 (44%)
Solitary	6	6 (100%)	3	1 (33%)
Multiple (2–3)	4	2 (50%)	8	5 (63%)
Multiple (≥ 4)	4	2 (50%)	7	2 (29%)
Lung	9	5 (56%)	14	3 (21%)
Solitary	5	4 (80%)	2	1 (50%)
Multiple (2–3)	2	1 (50%)	5	1 (20%)
Multiple (≥ 4)	2	0	7	1 (14%)
Uni-lobe	6	5 (83%)	4	1 (25%)
Bi-lobe	3	0	10	2 (20%)
Other organs	10	4 (40%)	16	5 (31%)

**Table 4:** Recurrence in single, two, more than 2 organs and salvage surgery.

Status of recurrence	AN		Control	
	Cases of recurrence* (N = 23)	Cases of salvage surgery (n = 15)	Cases of recurrence* (n = 26)	Cases of salvage surgery (n = 11)
Isolated organ	14	11 (79%)	8	5 (63%)
Multiple organs				
2	9	4 (44%)	14	5 (36%)
≥ 3	0		4	1 (25%)

*: P = 0.0375 (χ^2^ = 6.564)

## Discussion

Resection of hepatic metastases increases survival in colorectal cancer, but the relapse rates are high and occur most commonly in the liver. A recent Memorial Sloan-Kettering Cancer Center (MSKCC) publication reported an analysis of poor prognostic factors and their impact on long-term survival and cure. Interestingly, the use of adjuvant HAI chemotherapy was associated with a higher 10-year survival compared with the rest of the patients [2]. Kemeny et al [10] reported that HAI and systemic therapy versus systemic therapy alone resulted in a 2-year survival (the primary endpoint) of 86% *vs* 72%, respectively (p = 0.03). The updated analyses revealed a 10-year survival of 41% and 27% and a median PFS of 31 and 17 months (p = 0.02), respectively [20].

Interest in epigenetic therapy using histone deacetylase inhibitors (HDAi) and DNA methyltransferase inhibitors in cancer treatment has been growing recently. Yeo W et al [21] reported that the HDAi Belinostat improved the partial response and stable disease rate from 2.4% to 45.2% in patients with unresectable hepatocellular carcinoma. Wagner J et al [22] noted that epigenetic changes are potentially reversible and that they are amenable to pharmacological interventions. Wagner J and Qui T [22,23] pointed out that although data from clinical trials indicates that little or no clinical benefit was observed in solid tumor malignancies with HDAi alone, remarkably, synergistic effects been observed from combination therapy with HDAi and different chemotherapeutics, other epigenetic drugs or targeted agents as well as with radiotherapy.

Sodium phenylbutyrate (PB) is an HDAi which has been approved for urea cycle disorders and now being investigated extensively in cancer field [24]. Sung MW et al [25] reported the feasibility of using a combination of 5-FU and PB as cytotoxic-differentiation therapy in advanced colon cancer, showing disease stabilization in 75% (3/4) of patients.

Sodium phenylbutyrate is converted to phenylacetate (PA) and conjugated with glutamine to form phenylacetylglutammine (PG) *in vivo* [24]. PB and sodium PA which is one of main ingredients of antineoplaston AS2–1, induce cytostasis, differentiation,and apoptosis in the tumour cells of various hematological malignancies and solid tumors, including glioma, neuroblastoma, leukemia, and adenocarcinomas of the breast, colon, and lung, by several cellular mechanisms [26–29]. PB and PA regulate gene expression through DNA-hypomethylation, inhibition of histone deacetylase, inhibition of protein isoprenylation, and glutamine depletion [28,30,31].

Antineoplastons A10-I (PG: metabolite of PA and isoPG) and AS2–1 (PG and PA) also induce cytostasis, differentiation, apoptosis, and G1 cell arrest in various tumor cell lines *in vitro* [14,15,27], and antineoplaston AS2–1 induces DNA-hypomethylation [32].

The results reported here show that the CSS rate in the group treated with antineoplastons was significantly higher than that in the control group, although the RFS were similar between the two groups. Recurrence rate in each organ such as the liver and lung in the AN arm also tended to be lower compared with those in the control arm. RFS after liver resection may not fully relate to CSS and overall survivals. Some patients who progressed after liver resection exhibited solitary recurrence in residual liver tissue or had one small lung metastasis which could be resected; therefore, their survival rate remains high. Of interest, the majority of recurrent tumors in the AN arm tended to be eligible for salvage surgery. Conversely, in the control arm widespread and unresectable multiple recurrences with multiple liver and/or lung metastases or intra-abdominal metastases occurred which were associated with a decreased survival rate. All patients received the same standards of treatment and care during the course of the study provided by the same medical team and being followed-up completely. We continued to observe CSS up to 10 years because Tomlinson et al reported the 10-year survival as cure in 2007 [2]. The 10-year CSS rate was 35% in the AN arm and 29% in the control arm.

Buckner JC et al [33] reported the efficacy and toxicity of antineoplaston A10 and AS2–1, but did not confirm the antitumor efficacy with recurrent glioma. They noted the adverse effects of intermittent i.v. injections of antineoplaston A10 and AS2–1 included reversible somnolence, confusion and exacerbation of an underlying seizure disorder. Intermittent i.v. administration of antineoplaston A10 and AS2–1 as Buckner et al reported [33], may increase rapidly plasma concentration of phenylacetylglutamine or phenylacetate which can cause reversible neurological adverse effects such as somnolence, confusion, seizure, all due to the high osmolarity of the solution.

The main adverse effects of antineoplastons in our study with continuous infusion of antineoplaston A10 followed by oral administration of antineoplaston AS2–1 were drug-related symptoms such as fullness of the stomach and phlebitis.

In this study we observed 66 adverse events (AEs) in the AN arm and 51 in control arm. The difference in the number of AEs was mainly attributed to three AEs; fullness of the stomach (8 versus 2, p = 0.04), anorexia (8 versus 4) and phlebitis (6 versus 0, p = 0.024) in the AN and control arms, respectively. The statistical significance should be cautiously interpreted because multiple testing increases the probability of false positive findings. These AEs were not severe and other AEs showed no significant difference in occurrence and severity between two arms. We did not observe any neurological AEs such as hypernatremia, or severe bone marrow dysfunction in our study, but it would be important to bear in mind that these potential adverse effects may happen during the course of treatment.

This side-effect profile of antineoplastons with the dose we used in this study, showing a relatively low level of toxicity, offers another significant advantage when considering postoperative adjuvant therapy, as these results suggest that antineoplastons can be safely combined with conventional cytotoxic chemotherapeutics.

We delayed in publication of this study simply because we wished to observe the CSS up to 10 years and because we have a linguistic challenge in the preparation of manuscripts in a foreign language.

We conclude that in this study antineoplastons have demonstrated a promising level of efficacy against postoperative recurrence with a median CSS time of 67 months in the AN arm versus 39 months in the control arm, achieved via inhibition of early growth in metastatic tumors from colorectal cancer. The administration of antineoplaston AS2–1 may be clinically effective as a postoperative adjuvant therapy in liver metastasis from colorectal cancer. The results of this study have warranted the initiation of a new randomized, controlled clinical trial of adjuvant therapy using antineoplaston A10 and AS2–1 combined with HAI after hepatectomy in patients with colorectal metastasis to the liver.

This work was presented as an abstract at the 35^th^ EMSO congress in 2010 (Abstract No. 697). [34]

## Supporting Information

S1 CONSORT Checklist The CONSORT Checklist indicates the section that contains each item including randomization. (PDF)

S1 Protocol Description of the study protocol in English. (PDF)

S2 Protocol Description of the study protocol in Japanese. (PDF)

S1 Ethical Approval A copy of the ethical approval in Japanese by Kurume University School of Medicine dated on March 17, 1998. (PDF)
