# Supplementary material for: Randomized Phase II Study of 5-Fluorouracil Hepatic Arterial Infusion with or without Antineoplastons as an Adjuvant Therapy after Hepatectomy for Liver Metastases from Colorectal Cancer
Source: PLoS One. 2015 Mar 19;10(3):e0120064. doi: 10.1371/journal.pone.0120064 (PMC4366171; doi:10.1371/journal.pone.0120064)
Supplement: S1 Protocol — (PDF) [file pone.0120064.s002.pdf]

Protocol (English Translation)

Randomized Phase Ⅱ Study of Hepatic Arterial Infusion with or without Antineoplastons as Adjuvant Therapy after Hepatectomy for Liver Metastases from Colorectal Cancer(Version 4)

Principal Investigator: Professor Kazuo Shirouzu MD
Chairman of Department of Surgery
Kurume University School of Medicine
Chief Study Coordinator: Assistant Professor Yutaka Ogata MD
Department of Surgery
Kurume University School of Medicine
Associate Professor Hideaki Tsuda MD
Department of Anesthesiology
Kurume University School of Medicine

Place of Study conducted: Kurume University Hospital
67 Asahimachi Kurumeshi
Fukuokaken, Japan

Version 1: June 17, 1997
Version 2: February 1, 1998
Version 3: January 1, 2001
Version 4: February 5. 2003

1. Background and purpose of this study

Liver metastasis is a major limiting factor to the prognosis of colorectal cancer. Recently the progressive surgical removal of metastatic liver tumors becomes practical because of development of intraoperative ultrasound technique and improves survival rate when they are resectable.

Even in unresectable cases there are reports of efficacy of intra hepatic arterial infusion of chemotherapeutics (HAI), trans-arterial embolization (TAE) and microwave coagulation necrosis (MCN).

To contribute to better result after surgical removal of metastatic liver metastases it is inevitable to control metastasis in the residual liver and extra-hepatic metastasis especially to lungs.

We have tried to use intra- hepatic arterial infusion of 5-FU to control metastasis in the residual liver but the hepatic arterially infused 5-FU is not expected to work on extra-hepatic metastases because it is deactivated in liver. The systemic chemotherapy should be added to this situation but the application of cytotoxic chemotherapeutics in early postoperative stage is not practical at all from the point of their adverse effects.

Antineoplastons are naturally occurring peptides and amino-acid derivatives which control neoplastic growth and have been found in blood and urine under the concept of biochemical defense against cancer. They work on divert mechanism of action. Researchers reported its antagonism against chemical and viral carcinogen, competitive up take of tumor cells against l-glutamine which is nitrogen source to tumor cells which lead G1 arrest in tumor cells, induction of cell differentiation in tumor cells, deactivation of oncogenes, activation of tumor suppressor genes through the alteration in DNA methylation status. And most of all they are quite non-toxic.

Antineoplastons could be combined with other chemotherapy because of less toxicity and also restored effect of chemo-resistance in tumor cells could be expected by Antineoplastons.

Phase I toxicological clinical study conducted in Kurume University Hospital from 1988 showed only minor adverse effects such as skin rash, excess gas, rigid finger, headache, lowered cholesterol.

We experienced a case of long term survive from multiple liver metastases from colon cancer. This case motivated us to conduct this study to evaluate efficacy of Antineoplaston A10 and AS2-1 as adjuvant therapy after hepatectomy for liver metastases from colorectal cancer.

2. Protocol Summary

    1) Study Design: This is randomized phase II clinical trial

2) Purpose: Evaluation of anti-tumor efficacy against postoperative intra and extra hepatic metastasis and toxicity of Antineoplaston A10 and AS2-1 in colorectal cancer.
3) Eligibility: Eligible patients had histologically confirmed metastatic colorectal adenocarcinoma to the liver. Patients were treated with R0 resection of liver metastases and/or complete ablation by radio frequency interstitial ablation therapy between April 1998 and August 2004 at Kurume University Hospital.
4) Treatment: In control group, patients received HAI with 5-FU at a dose of 1000 mg/m^2 for 4 hours weekly or biweekly after hepatectomy until the cumulative dose reached up to 15,000 mg.

   In Antineoplaston group, Antineoplaston A10-Injection and AS2-1 were administered from day 15 after liver surgery in addition to HAI same as in control group with a starting dose of 30 g/day of intravenous A10-Injection using a pump system to maximum dose of 100 g/day for more than 3 days. After completion of the i.v. administration of A10-Injection for a week, 10 g/day of AS2-1 was administered orally for 1 year.
5) Primary Study Endpoints: The efficacy and safety endpoints will be

   Cause-specific survival rate, Disease free survival rate, Degree of recurrence, frequency, severity of adverse events.
6) Total number of patients: a total of 50 patients will be enrolled with 25 patients randomized to one of two treatment groups.
7) Duration of Study: The study will conduct from April 1998 to December 2004 and the prognosis of patients will be followed for at least 5 years.

3. Purpose of the study

To test efficacy and safety of Antineoplaston A10 and AS2-1 in adjuvant use in addition to HAI (5-FU) after hepatecomy for metastatic liver cancer from colon.

4. Eligibility

1) Inclusion Criteria

Patient who underwent curative hepatectomy or thermal ablation for liver metastases histologically confirmed as a metastasis from colon.

2) Exclusion Criteria

a) Patient who received other anti-tumor medication, differentiation inducer, radiotherapy at least in the recent 4 weeks.
b) Patient in severe major organ dysfunction.
c) Patient whose age over 75 years-old.
d) Patient whose ECOG physical status over 3.
e) Patient who do not tolerate HAI of 5-FU.
f) Patient who has extra-hepatic lesion or other type of cancer.
g) Patient who is pregnant or possibly pregnant.
h) Patient whose WBC less than 3,000/mm^3
i) Patient whose neutophile less than 1,500/mm^3
j) Patient whose platelet less than 75,000/mm^3
k) Patient whose GOT,GPT over 100 IU
l) Patient whose total bilirubin over 2.0 mg/dl
m) Patient whose creatinine over 1.5 mg/dl
n) Patient whom attendant investigator judges not appropriate to undergo this trial.

5. Informed Consent

It is the responsibility of the investigator to provide oral and written information about this study and obtain to obtain a sighed informed consent from each patient participating with free will in this study prior to enrollment.

In case of the patient who is unable to understand what this study about, informed consent should be obtain from a person representing patient benefit legally. In case of the patient whose age is under 20 years informed consent should be obtained from the patient and legal representative.

The following issues should be fully explained before getting informed consent.

1) The purpose and method of this study.
2) Expected efficacy and toxicity.
3) Other therapeutic modalities could be applied to the disease.
4) Patient do not get disadvantage when he(she) do not anticipate to this study.
5) Patient can decline participating to this study treatment anytime after he or she sign the informed consent.
6) Patient should not claim compensation for any result from this study.
7) Necessary consideration for protecting patient's human right.

Informed consent should be in document, clearly noted that signed by the patient him (her) self or legal representative and the name of attendant investigator

physician and the date of informed consent obtained.

6. Investigational Drugs

Antineoplaston A10 Injection

(4:phenylacetylglutamine+1:phenylacetylisoglutamine) (300mg/ml)

Antineoplaston AS2-1

(1:phenylacetylglutamine+4:phenylacetic acid) (500mg/capsule)

Provided by Burzynski Research Institute,INC.

7. Administration

Hepatic arterial infusion of 5-FU

5-FU is administered through a catheter instituted in hepatic artery, confirmed the position by angiogram, in early post-operative stage after hepatectomy either intermittently (1000 mg/m2/ for 4 hours/1-2 weeks) or continuously 180 mg/day. Continuous infusion of 5-FU may interrupt one week. Administration of 5-FU ends with total dose 15 g infused.

Antineoplaston A10 Injection and AS2-1

On 15th day after liver surgery Antineoplaston A10 Injection (30 g/day) starts to be infused through the catheter instituted in great vein and increase the dose up to maximum 100 g/day and keep this dose for 3 days. Then Antineoplaston AS2-1 10 g/day (at breakfast, lunch, dinner and before sleep) starts to be administered and continued for one year.

8. Randomization

Eligible patients are randomly assigned to receive systemic antineoplastons plus 5-FU HAI (AN arm) or 5-FU alone (control arm) by minimization method using number of metastases (1-3 vs >4) and presence or absence of prior extra-hepatic metastases which were removed completely at the time of surgery.

9. Drugs during the study period

No other anti-cancer drugs should be used during the study period.

No limitation of use of anti-histamine or digestive medication is applied.

10. Therapy during the study period

Chemotherapy, immunotherapy and radiotherapy are not conducted during the study period. When any of those therapies has conducted with un-evitable reason the content of the conducted therapy, when it has conducted and details of course history should be recorded.

11. Observation and time schedule for tests

1) Patient information before the study treatment
Name of patient (initial), gender, age (date of birth), in-patient or out-patient
Clinical diagnosis, histological diagnosis, clinical stage, physical status, disease history before treatment, past history of disease and allergy

2) Previous treatment to the present disease

3) Time schedule of observation and tests

	control	1month	2month	3month	6month	9month	12 month	Every 3 month
AS2-1		→	→	→	→	→	→	→
Symptoms	○	○	○	○	○	○	○	○
Recurrence(CT)	○	○	○	○	○	○	○	○
Clinical tests	○	○	○	○	○	○	○	○
Tumor marker	○	○		○	○	○	○	○
Frozen Serum	○							
E.C.G.	○						○	

4) Subjective and objective symptoms
Subjective and objective symptoms related to treatment are graded according to the Common Terminology Criteria for Adverse Events (CTCAE version 2 provided by Japanese Society of Cancer Therapy) for toxicity and adverse

events reporting.

Any abnormal symptom should be graded in relation of treatment into 4 categories: 1. obviously related 2, probably related. 3. possibly related 4. Not related

5) Search for recurrence and metastasis

Ultrasound sonography, CT to search recurrence and metastasis, should be conducted at least once every 3 months in post-operative 2 years and thereafter at least once every 6 months up to 5 years.

The location, number, size and extent of recurrence and metastasis should be examined and recorded.

6) Clinical tests and physical examination

The following clinical tests and physical examination are carried out pretreatment, one month after treatment and thereafter every 3 months. Any abnormal finding of these tests and examination found after treatment should be graded to 4 categories: 1. obviously related 2, probably related. 3. possibly related 4. Not related

a) Physical examination: blood pressure, heart rate, Performance Status
b) Hematology: WBC count, fraction of WBC, RBC count, Hb, Ht, Platelet count
c) Serological tests: GOT, GPT, ALP, LDH, γGTP, ChE, Total bilirubin, direct bilirubin, total protein, albumin, total cholesterol
   BUN, creatinine, uric acid, Na, K, Cl, Ca, P, blood glucose amylases, ICG, HPT, PT
d) Urological tests: protein, glucose, urobilinogen
e) Electrocardiogram

7) Tumor marker

CEA should be measured prior to treatment, one month after treatment and thereafter once every 3 months.

8) Preserve frozen serum

The patient's serum is preserved frozen for measurement of newly developed tumor marker or additional tests necessary in future.

9) Adverse events

Adverse events other than subjective and objective symptoms happening during the study period should be graded in severity according to CTCAE version 2 and recorded with date, time of event and treatment and response.

10) Diary

Patient is requested to record any abnormal symptom, frequency of symptom, duration of symptom he (she) feels in diary.

12. Withdrawal criteria

The study should interrupt in cases with following reasons

1) Progressive deterioration of symptom from disease
2) Deterioration of general condition from complication
3) Appearance of unexpected event
4) Unable to administer testing drugs due to adverse effects
5) Patient or family want to withdraw from the study
6) Unable to follow patient
7) When attendant investigator judge not to continue the study

In the interrupted cases, clinical observation, physical examination and clinical tests should continue as scheduled to evaluate the efficacy and toxicity of testing drugs.

In the interrupted cases who have not visited hospital, an effort should be paid to follow up the patients to clarify the prognosis and the reason why not visiting hospital.

13. Registered patients in the study are categorized into appropriate, inappropriate, perfectly treated, imperfectly treated and appropriate perfectly treated by the standard of Japanese Cancer Patient Follow up Committee in Japanese Society of Cancer Therapy.

14. Evaluation of efficacy

Efficacy of the testing drug should be evaluated by comparing cause specific survival, time to intra-hepatic recurrence, degree of extension of recurrence and time to extra-hepatic recurrence between Antineoplatstons treated group and control group.

15. Serious adverse effects during the study should be reported immediately to the

safety committee and treated properly. The safety committee of this study consists of principal investigator and study investigators. The safety committee should be open to patient's contact 24 hours a day.

16. Follow up

Treatment of patient disease after the study completed will be depending on attendant physician's decision, the prognosis of patient will be followed up to 5 years after study treatment.

17. Sample size

Statistical consideration for obtaining significance at 5 years survival needs 25 patients in each group making total 50 patients.

18. Time to start the study

The study starts from April, 1998 for 10 years.

19. End of the study

The study will ends enrollment as soon as 50 patients finish the study treatment.

20. Recording

1) Recording should be written in black
2) Correction of description must be done by X mark or two lines with signature who made correction. The date and reason for correction must be noted if the correction is major and serious.

21. Management of testing drugs

Testing drugs are managed by a study investigator who takes care of drug management.

22. Organization of conducting this study

Lower Gastrointestinal Group in Department of Surgery, Kurume University, School of Medicine

Antineoplaston Study Group in Kurume University, School of Medicine
