# Supplementary material for: Randomized Phase II Study of 5-Fluorouracil Hepatic Arterial Infusion with or without Antineoplastons as an Adjuvant Therapy after Hepatectomy for Liver Metastases from Colorectal Cancer
Source: PLoS One. 2015 Mar 19;10(3):e0120064. doi: 10.1371/journal.pone.0120064 (PMC4366171; doi:10.1371/journal.pone.0120064)
Supplement: S2 Protocol — (PDF) [file pone.0120064.s003.pdf]

試験計画書

大腸癌肝転移症例におけるアンチネオプラストン A10 及び AS2-1 の肝切除症例における残肝、

肝外再発予防効果及び副作用に関するランダム化第 II 相臨床試験（ver.4）

試験総括医師

久留米大学医学部　外科教授　　白水　和雄

講師　　緒方　　裕

麻酔科助教授　　津田　英照

試験実施施設

久留米大学病院

作成年月日：　1997年6月17日（ver.1）
1998年2月　1日（ver.2）
2001年1月18日（ver.3）
2003年2月　5日（ver.4）

1.　本試験計画の背景と目的

肝転移は大腸癌の予後を規定する最も大きな因子である。近年、肝転移に対して積極的な外科的切除が行われるようになり良好な成績も報告されている。また、切除不能な肝転移に対しては肝動注化学療法、TAE（Trans Arterial Embolization）、あるいはMCN（Microwave Coagulation Necrosis）などの有効性が報告されている。しかし、さらに肝転移症例の成績を向上させるためには肝切除例においては残肝再発と肝外病変のコントロール、肝切除不能例では肝動注化学療法による肝内腫瘍のコントロール、が可能な症例では肝外病変、とくに肺転移のコントロールが重要な課題である。

我々は肝切除後の残肝再発防止を目的として肝動注化学療法を導入しその評価を行ってきたが、5-FUを用いた肝動注化学療法では薬剤が肝臓内で不活性化されるため肝外病変化に対する効果は期待できず、肝外再発の防止には有効な全身的化学療法を付加すべきと考えている。しかし、肝切除後早期に肝動注化学療法に加えて強力な全身的化学療法を併用することは副作用の面から考えて現実的ではない。

Biochemical defense mechanism against cancer　の概念のもとヒトの血中及び尿中から抽出されたpeptideであるantineoplastonsは、carcinogenとの拮抗作用、l-glutamineとの拮抗作用による腫瘍細胞のG_1期での成長停止、ras family oncogeneの産物p-21の不活性化による腫瘍細胞増殖抑制効果、DNA methylationを介した癌抑制遺伝子の活性化、腫瘍細胞の分化誘導作用など多彩な作用機序を持つと共にその極めて低い毒性という特徴から従来の化学療法との併用が可能であり、かつ前治療により薬剤耐性になった腫瘍細胞への効果も期待できる。平成元年から久留米大学病院で実施されたAntineoplaston A10,AS2-1 の第Ⅰ相毒性試験では皮疹、腹部膨満、手の指の強ばり、頭痛、コレステロールの低下など軽微なものであった。腫瘍抑制効果はとくに肝臓腫瘍においてみられ大腸癌の多発肝臓転移で3年以上quality of life　を保ったcancer free症例を経験している。

そこで、本試験では肝動注化学療法に付加する Antineoplaston A10,AS2-1 の大腸癌肝転移肝切除例（ラジオ波焼灼以下 RFA を含む）における術後補助療法としての有効性および安全性を検討する。

2.　試験の要約

1).　試験の段階：無作為割付第 II 相臨床試験

2).　試験の目的：Antineoplaston A10.AS2-1 の大腸癌肝臓転移症例における抗腫瘍効果、肝内外再発予防効果並びに一般毒性の検索

3).　対象：組織学的に大腸癌肝転移と確認され、治癒的肝切除（RFA 併用を含む）が施行された症例

4).　治療法：

Antineoplaston+肝動注（Antineoplaston）群：

肝切除後 Antineoplaston A10（300mg/ml）を 30g-100g/day を持続的に投与する静脈注入（30g/day より開始し、速やかに最大投与量 100 g/day まで増量する、最大量を 3 日間投与して終了後、Antineoplaston AS2-1（500mg/錠）20 錠/日を術後 1 年間内服する。また、肝切除後可及的早期に肝動脈にカテーテルを留置し Angiography で血流分布を確認後 5-FU を総計 15g 投与する。

肝動注単独（コントロール）群：

肝切除後可及的早期に肝動脈にカテーテルを留置し Angiography で血流分布を確認後 5-FU を総計 15g 投与する。

5). 主たる評価項目:癌特異的生存率、無再発生存率、再発度、有害事象
6). 目的症例数:Antineoplaston 群 25 例、コントロール群 25 例
7). 試験実施予定期間:平成 10 年 4 月から平成 16 年 12 月まで、ただし予後調査は治療開始後 5 年まで追跡調査を行う。

3. 目的

Antineoplaston A10.AS2-1 の大腸癌肝臓転移治療肝切除例における有効性および安全性を検討する。

4. 対象患者

1). 選択基準
組織的に大腸癌の肝転移と確認され外科的に治癒的肝臓切除(RFA 併用可)が行われた症例
2). 除外基準
a). 他の抗腫瘍薬、分化誘導薬、放射線治療を少なくとも最近 4 週間以内に受けている者
b). 重篤な多臓器障害をもつ者
c). 75 歳以上の者
d). 術前、治療開始前の ECOG の PS が 3 以上の症例
e). 肝動脈 5-FU 注入に耐えられない者
f). 肝外病変、他の重複癌のある者
g). 妊娠または妊娠の可能性がある者
h). 白血球数が 3000/mm3 未満の者
i). 好中球数が 1500/mm3 未満の者
j). 血小板数が 75000/mm3 未満の者
k). GOT,GPT が 100IU 以上の者
l). Total Bilirubin が 2.0mg/dl 以上の者
m). Creatinine が 2.0mg/dl 以上の者
n). 試験担当医師が不適当と判断した者

5. 患者の同意

試験の開始に当たり、試験担当医は本試験の内容を被験者に説明し、試験への参加について文書で自由意志による同意を得るものとする。
被験者本人の同意が得ることが困難な場合には、家族、法定代理人など被験者の最善の利益を測りうる人から同意を得る者とする。未成年者を被験者とせざるを得ない場合は代理人及び未成年者が理解能力を有する場合は本人の同意も得る。
同意を得るに当たり下記事項について被験者に説明するものとする。
1). 本試験の目的及び方法
2). 予期される効果及び危険性
3). 当該疾患に対する他の治療法の有無及びその内容
4). 被験者が試験の参加に同意しない場合であっても不利益を受けないこと
5). 被験者が試験の参加に同意した場合でも随時これを撤回できること

6). 被験者が試験中に受けた健康被害を求償できないこと

7). その他被験者の人権の保護に関し必要な事項

同意取得の記録として症例記録同意取得欄に

１. 文書で同意 ２. 本人か代理人（続柄）の別 ３. 同意取得医師名 ４. 取得年月日 を記載する。

6. 試験薬剤

Antineoplaston A10 Injection(4:pheny1lacetylglutamine+1:phenylacetylisoglutamine)(300mg/ml)

Antineoplaston AS2-1(1:phenylacetylglutamine+4:phenylacetic acid)(500mg/capsule)

Burzynski Research Institute INC.提供

7. 投与方法

肝動注

肝切除後可及的早期に肝動脈にカテーテルを留置しAngiographyで血流分布を確認後 5-FUを 1000mg/m^2/1-2 週毎の大量間欠投与（4 時間infusion）または 180mg//m^2/dayで持続肝動脈注入を行う。ただし持続肝動脈注入は 1 週毎の休薬期間を置いても差し支えない。動注は 5-FUの総量が 15gで終了とする。

Antineoplaston

IVH カテーテルより Antineoplaston A10 Injection を 30g/day より開始し、速やかに最大投与量 100 gr/day まで増量する、最大量を 3 日間投与して終了する。引き続き Antineoplaston AS2-1（500mg）20 錠を朝、昼、夕食及び就寝前に 4 回分服する。投与期間は 1 年間とする。

8. 患者振り分け

細小化法（肝外病変の有無、肝転移個数 3 個以下と 4 個以上）により Antineoplaston 群とコントロール群（動注単独群）に無作為に割付ける

9. 併用薬剤

本試験中は他の抗腫瘍剤は使用しない。

本試験中に必要と思われる抗ヒスタミン、消化剤などは制限しない。

10. 併用療法

本試験中は原則として化学療法、免疫療法、放射線治療などを併用しない。

やむを得ず行った場合は治療内容、実施時期を症例記録に記入する。

11. 観察：検査項目およびその時期

1). 患者背景

患者氏名（イニシアル）、性別、年齢、入院：外来の別。臨床診断名

組織的診断名、臨床病期、Physical Status、本治療開始前までの経過概要

合併症、既往症、アレルギーの既往歴などを記入する。

2). 前治療

前治療の有無について調査し、前治療が行われていた場合その内容を記録する。

3). 観察：検査事項と実施時期一覧表

	前	1 月	2 月	3 月	6 月	9 月	12 月	3 月毎
AS2-1 内服		●	—	—	—	—	▶	
症状	○	○	○	○	○	○	○	○
再発検索（CT）	○	○	○	○	○	○	○	○
臨床検査	○	○	○	○	○	○	○	○
腫瘍マーカー	○	○		○	○	○	○	○
血清凍結保存	○							
心電図	○						○	

4). 自、他覚症状

自、他覚症状については「固形癌化学療法直接効果判定基準(CTCAE vol.2)の副作用記載様式」に従って重症度を判定する。異常所見が認められた場合、本薬剤との関連性を「１：明らかに関連あり」、「２：関連性が高い」、「３：関連性がありうる」、「４：関連なし」の 4 段階で判定する。

5). 再発の検索

肝切除後の状態、肝動注脈化学療法後の状態を、超音波 CT で検索 以後原則として術後 2 年までは 3 ヶ月毎に、以後術後 5 年までは 6 ヶ月毎に CT にて再発の有無を評価する。

再発、転移が認められた場合は超音波検査、CT で再発、転移の部位、大きさ、数及び様式を観察する。

6). 臨床検査（一般検査、血液検査、血清生化学検査、尿検査）

下記の項目について投与前、投与後1ヶ月、3ヶ月毎に検査を実施する。試験薬の投与により異常変動が出現した場合はその後の経過及び本薬剤との関連性（１：明らかに関連あり、２：関連性が高い、３：関連性があり得る、４：関連なし）を記入する。

a). 一般検査：血圧、脈拍数、心電図、Performance Status

b). 血液検査：白血球、白血球分類、赤血球、ヘモグロビン、ヘマトクリット、血小板

c). 血清生化学検査：GOT,GPT,AL-P,LDH,γGTP,ChE,総ビリルビン、直接ビリルビン、血清総蛋白、アルブミン、蛋白分画、総コレステロール、BUN、クレアチニン、尿酸、Na,K,Cl,Ca,P、血糖、血清アミラーゼ、ICG,HPT,PT

d). 尿検査：蛋白、糖、ウロビリノーゲン

e). 心電図

7). 腫瘍マーカー

腫瘍マーカーCEA の測定は試験開始前、開始後 1 ヶ月、3 ヶ月毎に行う。

8). 血清の保存

新しい検査項目の追加、腫瘍マーカーの測定などを考慮し血清 1.0ml を 2 本に分注し凍結保存する。

9). 副作用

試験期間中に発現した副作用（本剤投与時の自他覚症状の異常所見以外）について発現日、重症度、処置、経過および本剤との関連性を記入する。

重症度は「固形癌化学療法直接効果判定基準の副作用の記載様式」に従って判定する。

10). 服用日誌

被験者に毎日の服用に際しその数、時間及び異常を感じた場合の症状、持続時間を日誌に記録してもらうものとする。

12. 中止：脱落基準

下記の理由により試験の継続が不可能となった場合はプロトコール中止または脱落例とする。

1). 症状の悪化
2). 合併症の悪化
3). 偶発性の出現
4). 副作用の発現で服用が困難
5). 患者または家族からの中止の申し出
6). 患者との連絡不能
7). その他試験担当医の判断

なお、中止とする場合は試験終了時と同様の観察、検査を行い可能な限り各評価を行う。試験期間に患者が来院しなくなった場合は追跡調査を行い来院しなくなった理由とその後の経過を明らかにする。

13. 症例の取り扱い

症例の取り扱いは癌治療学会の「癌患者追跡調査総論」における症例の取り扱い法に準じて、登録例、適格例、不適格例、完全治療例、不完全治療例、および適格完全治療例を定める。

14. 評価方法

肝切除後の癌特異的生存率、肝臓内再発までの時期、再発病巣の程度、肺外病変発生までの時期生存期間を Antineoplaston A10,AS2-1 使用群とコントロール群とで比較する。

15. 重篤な副作用が出現した場合の対応

本試験中、重篤な副作用が出現した場合には直ちに安全検討委員会に報告、適切な処置を講ずる。なお本試験安全検討委員会は試験総括医師を委員長として若干名の試験担当医師より構成、患者よりの連絡には 24 時間/日対応できる体制を整えておく。

16. 予後調査

試験終了後の治療については担当医師の判断に委ねるが予後の調査は治療開始後 5 年まで追跡調査する。

17. 目的症例数

統計学的に有意の比較を得るために Antineoplaston 投与群 25 例、対照群 25 例の合計 50 例を目標数として設定する。

18. 試験実施期間

平成 10 年 4 月より約 10 年間

19. 試験終了規則

目的症例数に達した場合は速やかに試験を終了する。

20. 症例記録記入における注意

1). 症例記録は黒のペンまたはボールペンで記入する。

2). 記入内容を訂正するときは訂正箇所を X 印または 2 本線で消した上から訂正印を捺印する。なお、訂正が大幅または重大な場合は訂正理由および訂正年月日を記入する。

21. 試験薬の管理

試験薬は本試験薬剤管理担当者が管理する。

22. 試験組織

久留米大学医学部外科下部消化管グループ
Antineoplaston 研究班
