# Supplementary material for: Randomized Phase II Study of 5-Fluorouracil Hepatic Arterial Infusion with or without Antineoplastons as an Adjuvant Therapy after Hepatectomy for Liver Metastases from Colorectal Cancer
Source: PLoS One. 2015 Mar 19;10(3):e0120064. doi: 10.1371/journal.pone.0120064 (PMC4366171; doi:10.1371/journal.pone.0120064)
Supplement: S1 Ethical Approval — (PDF) [file pone.0120064.s004.pdf]

（様式３）

審査結果通知書

平成１０年　３月１7日

申請者
白水　和雄　　　殿

久留米大学
医学部長　　森　松　　　稔 ㊞

研究番号　９７１７

研究題目　大腸癌肝転移症例におけるｱﾝﾁﾈｵﾌﾟﾗｽﾄﾝＡ１０及びＡＳ２－１の肝切除後の残肝、肝外再発予防効果並びに肝切除不能症例における抗腫瘍効果、肝外再発予防効果、及び副作用に関する一般臨床試験

研究者　白水　和雄　（主任研究者）外科学
　　　　緒方　　裕　（分担研究者）外科学

上記 ○研究計画 / 公表申請 を平成１０年　２月２４日の久留米大学医学部倫理委員会審査結果をふまえ、下記のように判定したので通知します。

記

判定	承認する　○条件付きで承認する<br>変更を勧告する　承認しない　該当しない
条件又は変更に関する委員会の意見	本試験に要する資金の調達（寄付）対象から「被験者及びその家族等」を除外すること及び資金が不足する場合は外科学（白水教授）の研究費を充当する旨を「実施計画書」に明記する。
